# Membrane-Targeting Perylenylethynylphenols Inactivate Medically Important Coronaviruses via the Singlet Oxygen Photogeneration Mechanism

**DOI:** 10.3390/molecules28176278

**Published:** 2023-08-28

**Authors:** Kseniya A. Mariewskaya, Daniil A. Gvozdev, Alexey A. Chistov, Petra Straková, Ivana Huvarová, Pavel Svoboda, Jan Kotouček, Nikita M. Ivanov, Maxim S. Krasilnikov, Mikhail Y. Zhitlov, Alexandra M. Pak, Igor E. Mikhnovets, Timofei D. Nikitin, Vladimir A. Korshun, Vera A. Alferova, Josef Mašek, Daniel Růžek, Luděk Eyer, Alexey V. Ustinov

**Affiliations:** 1 Shemyakin-Ovchinnikov Institute of Bioorganic Chemistry, Miklukho-Maklaya 16/10, 117997 Moscow, Russia; mariewskaya.k@gmail.com (K.A.M.); dobr14@yandex.ru (A.A.C.); nm-ivanov@live.com (N.M.I.); makrlist@gmail.com (M.S.K.); droplbox38@gmail.com (M.Y.Z.); alexunnipark@gmail.com (A.M.P.); mikhnovets.igor@gmail.com (I.E.M.); timanikitin36@gmail.com (T.D.N.); alferovava@gmail.com (V.A.A.); alex@lumiprobe.com (A.V.U.); 2 Department of Biology, Lomonosov Moscow State University, Leninskie Gory 1-12, 119234 Moscow, Russia; danil131054@mail.ru; 3 Laboratory of Emerging Viral Diseases, Veterinary Research Institute, Hudcova 296/70, CZ-621 00 Brno, Czech Republic; petra.strakova@vri.cz (P.S.); ivana.huvarova@vri.cz (I.H.); pavel.svoboda@vri.cz (P.S.); ruzekd@paru.cas.cz (D.R.); 4 Institute of Parasitology, Biology Centre of the Czech Academy of Sciences, Branišovská 1160/31, CZ-370 05 České Budějovice, Czech Republic; 5 Department of Experimental Biology, Faculty of Science, Masaryk University, Kamenice 753/5, CZ-625 00 Brno, Czech Republic; 6 Department of Pharmacology and Pharmacy, Faculty of Veterinary Medicine, University of Veterinary Sciences Brno, Palackého tř. 1946/1, CZ-612 42 Brno, Czech Republic; 7 Department of Pharmacology and Toxicology, Veterinary Research Institute, Hudcova 296/70, CZ-621 00 Brno, Czech Republic; kotoucek@vri.cz (J.K.); josef.masek@vri.cz (J.M.); 8 Department of Chemistry, Lomonosov Moscow State University, Leninskie Gory 1-3, 119991 Moscow, Russia

**Keywords:** perylene, photosensitizers, antivirals, singlet oxygen, SARS-CoV-2

## Abstract

Perylenylethynyl derivatives have been recognized as broad-spectrum antivirals that target the lipid envelope of enveloped viruses. In this study, we present novel perylenylethynylphenols that exhibit nanomolar or submicromolar antiviral activity against Severe Acute Respiratory Syndrome Coronavirus-2 (SARS-CoV-2) and feline infectious peritonitis virus (FIPV) in vitro. Perylenylethynylphenols incorporate into viral and cellular membranes and block the entry of the virus into the host cell. Furthermore, these compounds demonstrate an ability to generate singlet oxygen when exposed to visible light. The rate of singlet oxygen production is positively correlated with antiviral activity, confirming that the inhibition of fusion is primarily due to singlet-oxygen-induced damage to the viral envelope. The unique combination of a shape that affords affinity to the lipid bilayer and the capacity to generate singlet oxygen makes perylenylethynylphenols highly effective scaffolds against enveloped viruses. The anticoronaviral activity of perylenylethynylphenols is strictly light-dependent and disappears in the absence of daylight (under red light). Moreover, these compounds exhibit negligible cytotoxicity, highlighting their significant potential for further exploration of the precise antiviral mechanism and the broader scope and limitations of this compound class.

## 1. Introduction

The *Coronaviridae* family includes numerous medically significant viral pathogens, as evidenced by the recent outbreak of SARS-CoV-2 (Severe Acute Respiratory Syndrome Coronavirus-2), the causative agent of the COVID-19 pandemic, which has resulted in over 6 million deaths reported to date. In addition, Feline Coronavirus (FCoV), also called feline infectious peritonitis virus (FIPV), which causes lethal infections in domestic cats and wild felines, is currently an intensively researched veterinary pathogen. The treatment of diseases caused by members of the *Coronaviridae* family continues to be a challenge, making the development of new effective drugs against acute coronavirus infections an important research priority [1,2,3,4].

The versatile and light-dependent antiviral activity of perylene-related hypocrellin- [5,6,7,8,9] and hypericin-type [10,11,12,13,14] compounds has been known for decades. The other main broad-spectrum membrane-targeting perylene compounds are perylenylethynyl derivatives. Initially, their mechanism was believed to primarily involve biophysical fusion inhibition [15,16,17], however, more recent evidence has highlighted the significant role of photosensitization in their mode of action [18,19,20]. These compounds are efficient photogenerators of singlet oxygen (^1^O_2_), which exerts considerable damage on the unsaturated components of the lipid bilayer, particularly when the photosensitizer and double bonds are in close proximity (referred to as the contact-dependent pathway) [21]. This effect is based on ene reaction-driven lipid oxidation, followed by the cleavage of the C-C bond [21,22]. The ^1^O_2_-induced formation of short polar lipids dramatically changes the membrane rheology, and, in the case of virion envelopes, their ability to fuse with the host cellular membrane [23,24]. Generally, membrane-targeting ^1^O_2_-generating compounds show remarkable antiviral effects in vitro, e.g., arylidene rhodanines (LJ compounds) [25,26,27,28], methylene blue [29,30,31,32], riboflavin derivatives [33], iodinated BODIPY [34], fullerene derivatives [35], phthalocyanines [36,37,38], pheophorbide *a* [39] and other porphyrins [40,41,42], AIE compounds [43], alkyl Rose Bengal derivatives [44], and a genetically encoded photosensitizer [45].

**Figure 1:**
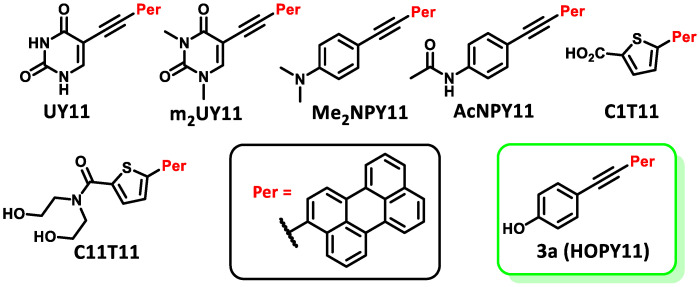
Recently studied perylene antivirals and the parent compound for this study (**HOPY11**).

Perylene [46,47,48] and its derivatives [19,49,50,51,52,53,54] have been recognized as singlet oxygen photogenerators. Recently, we determined the quantum yields of ^1^O_2_ generation in methanol for several (het)arylethynylperylenes and thienylperylenes, which have shown activity against SARS-CoV-2 [20]. Non-nucleoside perylene antivirals are amphipathic compounds that consist of a lipophilic perylene residue and polar functional groups (Figure 1). They are somewhat soluble in aqueous buffers containing a few percent of DMSO, likely forming micelles. The antiviral activity of these compounds is influenced by various factors, impacting not only their capacity to generate singlet oxygen ^1^O_2_, but also their ability to penetrate lipid membranes [20]. The ability of perylene antivirals to penetrate the membrane is strongly influenced by the shape and amphipathicity of the molecule: compounds with well-balanced amphipathicity had strong affinity for membranes and high anitiviral activity, whereas compounds with increased polarity or hydrophobicity were less effective or inactive [20].

**Figure 2:**
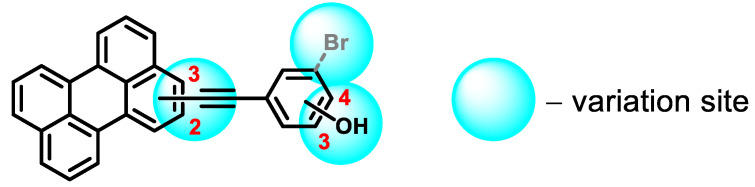
Structural design of phenol-conjugated perylenylethynyl derivatives.

During our investigation of perylene nucleosides, we synthesized the precursor compound, phenol **3a** [55]. Given its structural resemblance to other perylene antivirals (Figure 1), we decided to evaluate its antiviral activity, which yielded promising results. Considering the straightforward synthesis process, we proceeded to prepare a small series of similar compounds with modifications in the hydroxyl position, the perylene substitution position, and the inclusion of a heavy atom (bromine) into the molecule (Figure 2).

Our objective was to investigate spectral properties and singlet oxygen (^1^O_2_) generation ability of the synthesized perylenylethynylphenols and to evaluate their cytotoxicity and antiviral activity against two medically important coronaviruses, SARS-CoV-2 and FIPV. In addition, we elucidated the mechanism of their antiviral activity and demonstrated their specific interaction with viral envelopes and cellular and liposomal membranes. Finally, we have shown that the antiviral activity of perylenylethynylphenols is strictly light-dependent and is completely eliminated in the absence of excitation light or daylight. This work follows our previous larger study of the anti-SARS-CoV activities of perylene-based compounds [20], explains their mechanisms of anti-coronaviral activities in more detail, and shows that other modifications of the perylenylethynyl scaffold can provide compounds with improved biological properties.

## 2. Results and Discussion

### 2.1. Synthesis

Our objective was to develop novel compounds based on perylene with the following features: (i) an extended π-system, where the aryl component is connected to the dye via an ethynyl bridge, (ii) a hydrophilic group and (iii) a “heavy atom”. By expanding the π-system, the lipophilic portion of the photosensitizer increases in length, enabling deeper penetration into the viral envelope and facilitating oxidation by singlet oxygen. The hydrophilic group not only enhances compound solubility, but also influences the dye’s insertion into the lipid bilayer. Introducing a bromine atom into compounds **3c** and **3f** allowed us to examine the impact of a “heavy atom” in the structure of perylenylethynylphenols on the photophysical and biological properties.

**Scheme 1:**
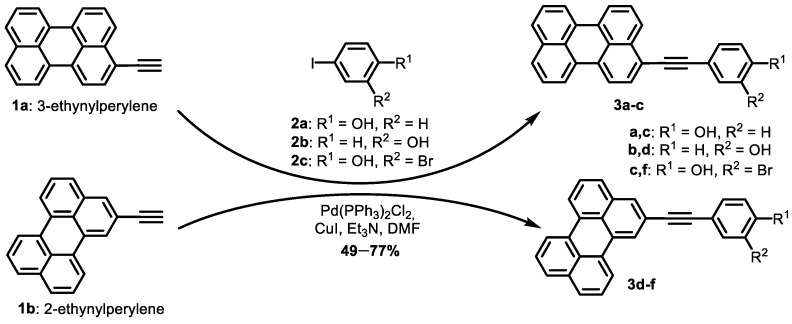
Synthesis of perylenylethynylphenols.

A series of novel perylene-based compounds was synthesized using a Sonogashira reaction (Scheme 1). The starting compounds included 2- and 3-ethynylperylenes **1a**,**b** and various iodoarenes **2a**–**c**. The reaction was performed under an inert atmosphere and heating (80 °C for 12 h). Compound **3a** was obtained using a previously reported method [55]. To remove residual DMF and catalyst, the reaction mixture was extracted with ethyl acetate, water and EDTA. The obtained compounds were purified using column chromatography. In total, six new compounds were obtained with yields ranging from 49% to 77%, giving the desired compounds as colored crystals.

### 2.2. Spectral Properties and ^1^O_2_ Generation

**Figure 3:**
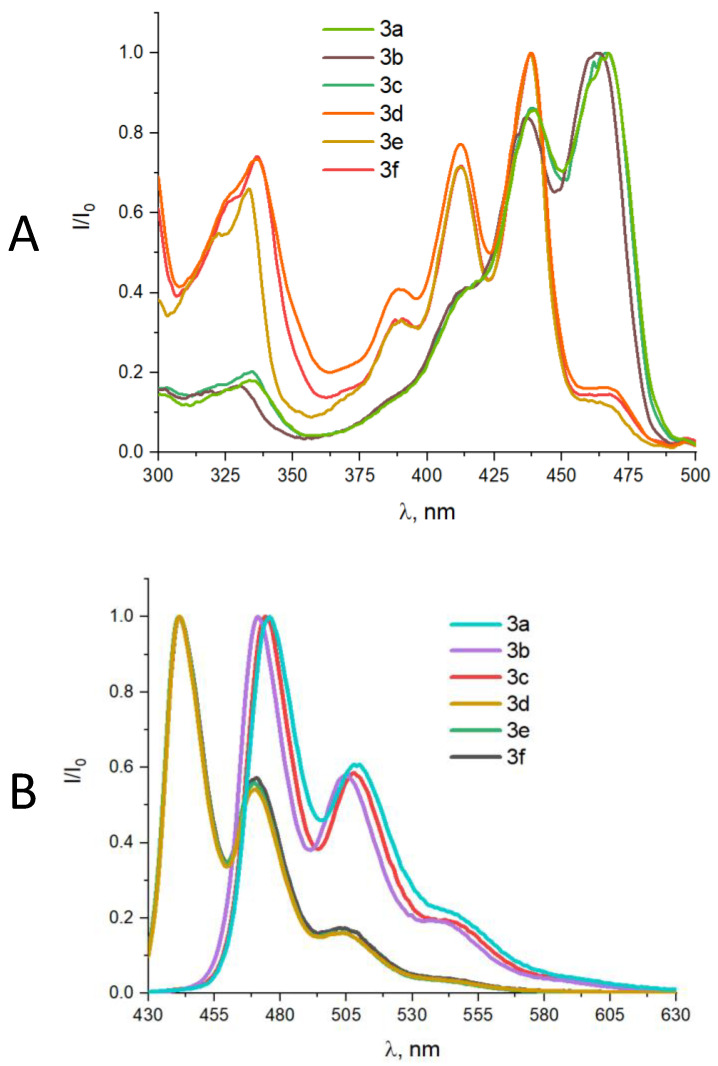
Normalized UV-Vis (**A**) and fluorescence (**B**) spectra of compounds **3a**–**f** in 96% EtOH.

The obtained perylene derivatives **3a**–**c** exhibited absorption maxima in the 463–466 nm range, while derivatives **3d**–**f** showed absorption at 438 nm (Figure 3). It is worth noting that perylene compounds substituted at the third position exhibit a bathochromic shift compared to perylenes substituted at the second position. Although aryl residues in diarylacetylenes are coplanar in their ground state, the rotation barrier is very low, and a number of conformations is present in solutions [56,57,58,59,60].

Compounds **3a**–**c** exhibited fluorescence maxima in the 472–476 nm range, while compounds **3d**–**f** showed fluorescence in the 440–442 nm range. A comparison of the absorption and fluorescence maxima between 3-ethynylperylene derivatives **3a**–**c** and 2-ethynylperylene derivatives **3d**–**f** revealed a red-shift of approximately 25–30 nm for the former (Table 1). The Stokes shift, representing the difference between the absorption and fluorescence wavelengths, was significantly lower for the 2-ethynylperylene compounds (2–4 nm) compared to the 3-ethynylperylene compounds (9–10 nm). Notably, due to the pronounced absorption peaks in the 430–470 nm range, perylene derivative molecules can undergo a transition from the excited singlet state to the triplet state, allowing for interaction with oxygen molecules to form singlet oxygen.

**Figure 4:**
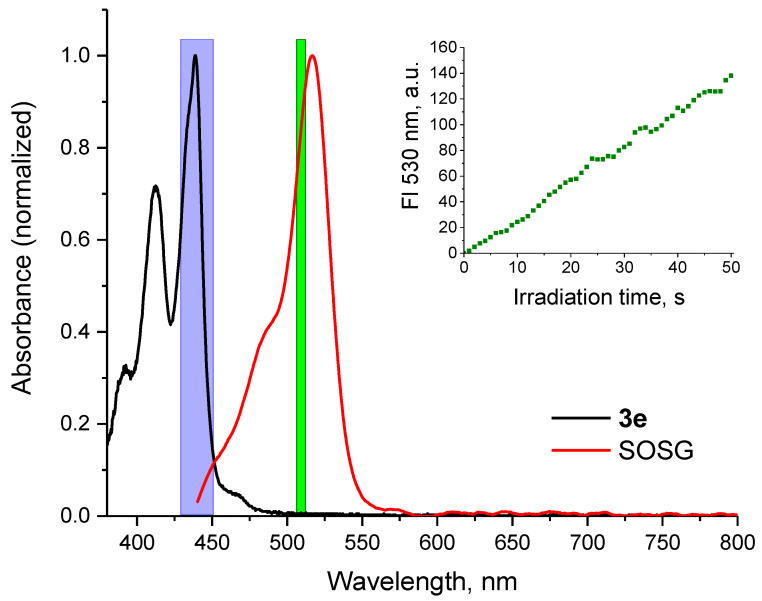
Normalized absorbance spectra of perylene **3e** and SOSG in methanol solution. Region of LED irradiation for singlet oxygen generation by perylene compounds is shown in blue, wavelength of SOSG excitation is shown in green. Inset: time course of SOSG fluorescence intensity in a reaction with ^1^O_2_ generated by perylene compound **3e** under blue light irradiation.

To determine the quantum yield of singlet oxygen generation, the absorption spectra of the photosensitizers and the SOSG indicator were measured (Figure 4). The rate of singlet oxygen generation was assessed by monitoring the fluorescence increase in SOSG (Figure 4, inset) and calculating the slope of the initial linear segment. The bleaching rate of the chemical trap under blue light irradiation in the absence of a photosensitizer was also considered.

Based on previous research on perylene antivirals [18,19,20], it is known that the photosensitizer generates singlet oxygen within the lipid bilayer of the virus envelope, resulting in its destruction and viral inactivation. Therefore, we conducted measurements to determine the quantum yield of singlet oxygen generation for compounds **3a**–**f** (Table 1).

### 2.3. Biological Studies

**Figure 5:**
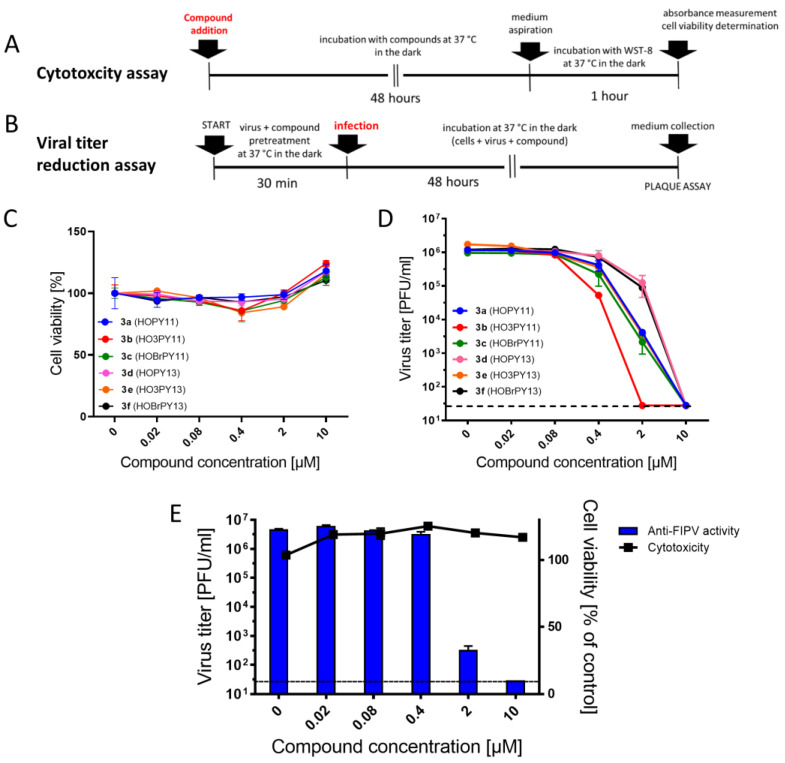
Cytotoxicity and anti-coronaviral activity of perylenylethynylphenols in vitro. (**A**) Schematic representation of the cytotoxicity assay using Cell Counting Kit-8 (Dojindo Molecular Technologies, Munich, Germany). (**B**) Schematic representation of the viral titer reduction assay. (**C**) Cytotoxicity of perylenylethynylphenols at the indicated concentrations for Vero cells. (**D**) Anti-SARS-CoV-2 activity of perylenylethynylphenols at the indicated concentrations in Vero cells. (**E**) Anti-FIPV activity of compound **3a** in CRFK cells. Data are expressed as the mean ± SD of two independent experiments, each performed in triplicate. The horizontal dashed line indicates the minimum detectable threshold of 1.44 log_10_ PFU/mL.

We first investigated the cytotoxicity and antiviral activity of perylenylethynylphenols **3a**–**f** against SARS-CoV-2 in Vero (African Green Monkey, adult kidney, epithelial) cells (Figure 5A–D). These initial experiments were performed under normal lighting (i.e., sample preparation in daylight and cultivation in the dark) (Figure 5A,B). Perylenylethynylphenols **3a**–**f** showed no cytotoxicity to Vero cells up to 10 µM (CC_50_ > 50 µM) when incubated with cells at 37 °C for 48 h (Figure 5C, Table 1). Interestingly, all compounds, when at the highest concentration tested (10 µM), caused a slight increase in the intensity of cellular metabolism, resulting in cell viability values above 100% (Figure 5C). However, no morphological changes were observed after culturing Vero cells with the tested compounds (up to 10 µM).

Given previously reported perylene derivatives with potent antiviral activity against TBEV and SARS-CoV-2 [6,7], we conducted further investigations to evaluate the activity of the newly synthesized compounds **3a**–**f** against SARS-CoV-2 in Vero cells. Derivatives of 3-ethynylperylene (**3a**–**c**) exhibited considerably higher activity against SARS-CoV-2 than derivatives of 2-ethynylperylene (**3d**–**f**) (Table 1, Figure 5D, Supplementary Table S1). This enhanced activity can be attributed to their stronger absorption in the 430–470 nm range and their structure being more favorable for positioning within the lipid bilayer, in close proximity to the unsaturated bonds of fatty acids.

Compound **3b**, which possesses a hydroxyl group in the *m*-position to ethynylperylene and lacks a bromine atom, exhibited the highest activity against SARS-CoV-2. On the other hand, compound **3c** displayed the lowest activity among the 3-ethynylperylene derivatives, and **3f** ranked among the lowest in the series (Table 1, Figure 5D). These results suggest that the presence of bromine in perylenylethynylphenols either reduces the antiviral activity of the compounds or has an insignificant effect on it. Considering the weak conjugation between the perylene and phenyl moieties of the molecule, it is likely that the bromine atom does not significantly influence the stabilization of the triplet state of the photosensitizer, thereby not affecting the yield of singlet oxygen generation, which directly correlates with antiviral activity.

Compound **3e**, among the derivatives of 2-ethynylperylene, showed the highest activity against the virus, while compound **3b** demonstrated the highest activity overall. This suggests that *m*-substituted peryleneylethynylphenols exhibit greater antiviral activity than *p*-substituted ones (Table 1, Figure 5D). Although the difference in the antiviral properties of perylene photosensitizers cannot be solely attributed to variations in their singlet oxygen generation capacity, a correlation can be observed when comparing the data on singlet oxygen generation with antiviral activity (Table 1). Specifically, structurally similar perylene derivatives with either 2- or 3-substitution tend to exhibit higher activity when they have a higher quantum yield of singlet oxygen generation. This indicates that some form of affinity to the lipid bilayer may also play a significant role as a prerequisite for achieving high antiviral activity [20].

**Figure 6:**
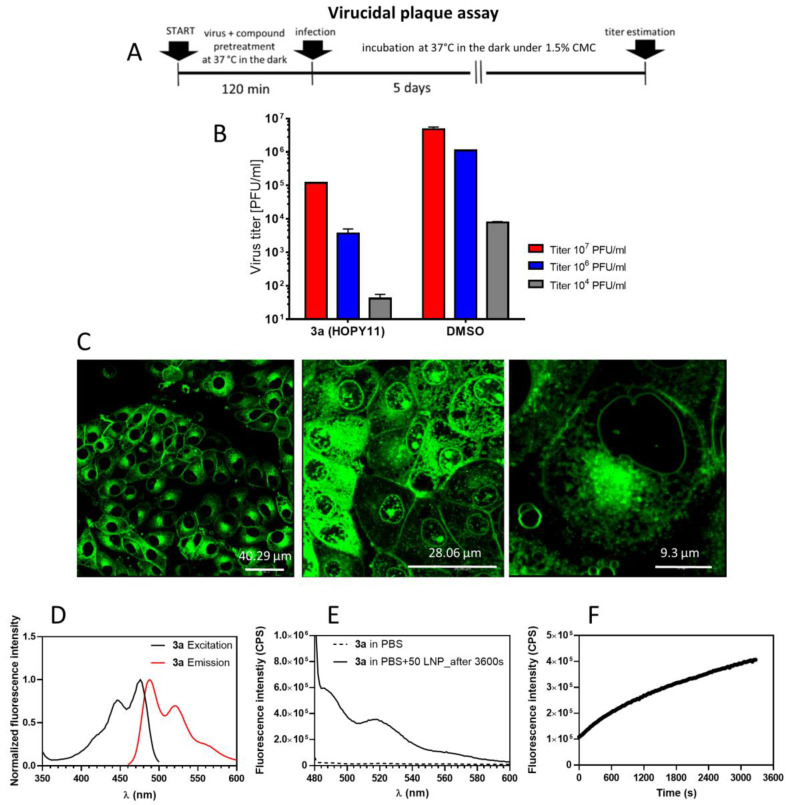
Interaction of compound **3a** with viral envelopes, cellular membranes and liposomal mebranes. (**A**) Demonstration of direct (virucidal) activity of **3a** against SARS-CoV-2 and its interaction with the viral envelope. Schematic representation of the experiment (virucidal plaque assay). (**B**) Quantification of the virucidal activity of **3a** using Vero E6 cells. The virus at the indicated titers was incubated with the compounds (10 μM) for 120 min. Viral titers were then quantified by plaque assays. (**C**) Penetration of **3a** into PS cells. Cells were seeded on slides for 24 h, then treated with **3a** (10 µM) and incubated for 1 h. Photomicrographs were taken using confocal microscopy. (**D**) Excitation and emission spectra of **3a** (10 µM) in DMSO. (**E**) Fluorescence spectra of free compound **3a** in PBS (10 µM, dashed line) and a mixture of **3a** and LPS in PBS. (**F**) Kinetics of the penetration of **3a** (10 µM) into liposomes, measured at 520 nm. Data are expressed as the mean ± SD of two independent experiments, each performed in triplicate.

Next, we selected a representative compound, **3a**, to further investigate the mechanism of anticoronaviral activity of perylenylethynylphenols. SARS-CoV-2 was pre-incubated with **3a** (10 µM) for 120 min (the mixture of compound and virus was prepared in daylight and incubated in the dark), and then the viability of compound-treated virus was estimated using a plaque assay (Figure 6A). Our mechanistic studies revealed that compound **3a** (and probably all synthesized perylenylethynylphenols) exhibited a direct (virucidal, virus-inactivating) effect on free viral particles and reduced/eliminated the viability of SARS-CoV-2 virions in the initial stages of the viral replication cycle. The decrease in titer after the treatment of SARS-CoV-2 with **3a** was strictly dependent on the starting virus titer (10^4^, 10^6^, and 10^7^ PFU/mL) and resulted in a decrease in viral titer of more than two orders of magnitude compared with control (Figure 6B). Thus, similar to other perylene-based antiviral agents [20], perylenylethynylphenols intercalate into viral membrane envelopes and act as blockers of the viral entry/fusion process. Blocking of virus–cell fusion was previously demonstrated for similar perylenylethynyl derivatives using a cell-based fusion assay [20].

Apart from the viral envelopes, perylenylethynylphenols also showed a strong affinity for cellular membranes; compound **3a** was extensively incorporated into the plasmatic membranes, nuclear envelopes and intracellular membranes (probably membranes of lysozomes or endosomes) of porcine stable kidney cells (PS), a model cell line suitable for efficient viusalisation of compound-cell interactions by confocal microscopy (Figure 6C). The incorporation of **3a** into cellular membranes is not surprising; both viral and cellular membranes share the same structural features and exhibit similar biophysical properties. Despite the intense incorporation of the compounds into cell membranes, we observed no signs of cytotoxicity or morphological abnormalities in PS cells treated with the compound (Figure 6C).

The affinity of **3a** to lipid membranes was further confirmed using a liposomal (protein-free) membrane model system (Figure 6D,E). Compound **3a** (10 µM) dissolved in PBS showed poor fluorescence capability; however, after the addition of **3a** to unilamellar liposomes (EPC/Chol of 70/30 mol%), the fluorescence significantly increased, indicating efficient penetration of **3a** into liposomal membranes (Figure 6D). The kinetics of liposome penetration of **3a** were measured as the steady-state fluorescence response at 520 nm, which reached maximal intensity of about 4 × 10^5^ CPE after the complete incubation period (3500 s) (Figure 6E).

Furthermore, we investigated the mechanism of antiviral activity of perylenylethynylphenols using FIPV, another member of the *Coronaviridae* family, which is an important veterinary pathogen. Under normal lighting (i.e., sample preparation in daylight and cultivation in the dark), compound **3a** showed nanomolar anti-FIPV potency (EC_50_ of 0.1958 µM, 95% CI of 0.1185–0.3234 µM) and no cytotoxicity to *Felis catus* kidney cortex (CRFK) cells (CC_50_ > 10 µM), a cell line commonly used for FIPV cultivation and FIPV-based plaque assays (Figure 5E).

**Figure 7:**
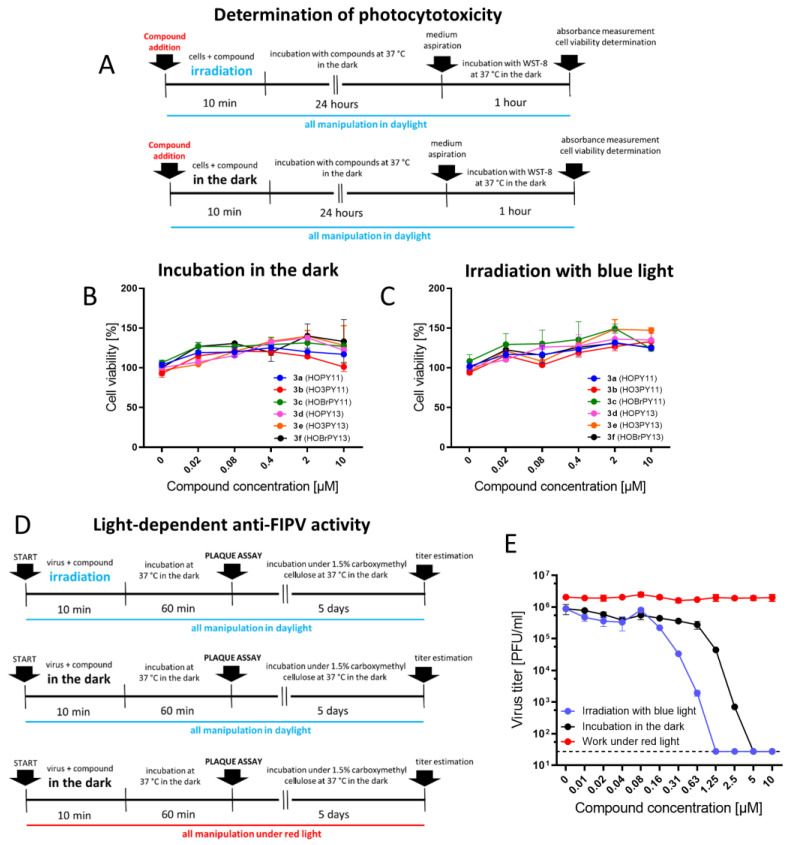
Light-dependent cytotoxicity and antiviral activity of perylenylethynylphenols. (**A**) Determination of photocytotoxicity (schematic representation of experiments). (**B**) Cytotoxicity of perylenylethynylphenols under normal light conditions (sample preparation in daylight, incubation of compounds with CRFK cells in the dark). (**C**) Cytotoxicity of perylenylethynylphenols after irradiation with blue light for 10 min at RT with LEDs (465–480 nm, 30 mW/cm^2^). (**D**) Light-dependent anti-FIPV activity of compound **3a** (schematic representation of the experiments). (**E**) FIPV was treated with **3a**, as described in (**D**), and the viability of the compound-treated virus was determined by plaque assays. Data are expressed as the mean ± SD of two independent experiments, each performed in triplicate. The horizontal dashed line indicates the minimum detectable threshold of 1.44 log_10_ PFU/mL.

Considering that perylenylethynylphenols are potent ^1^O_2_ photogenerators, we investigated their coronavirus inactivation activity after the irradiation of compound-pretreated FIPV with blue light (465–480 nm, with an approximate power density of 30 mW/cm^2^; note: the 465–480 nm wavelength is close to the excitation maxima of perylenylethynylphenols). In this experiment, (i) compound **3a** and the virus were mixed in daylight and irradiated with blue light for 10 min (Figure 7D, top panel), or (ii) the compound and virus were mixed in daylight and incubated in the dark for 10 min (Figure 7D, medium panel). Then, both samples (irradiated and non-irradiated) were incubated for 1 h at 37 °C in the dark, and the viability of FIPV was determined by a plaque assay. We observed a significant increase in anti-FIPV activity of **3a** after irradiation with blue light (Figure 7E, blue line), as compared with the control (non-irradiated sample, Figure 7E, black line). We speculate that the observed enhancement of antiviral (virus inactivation) activity of **3a** is due to increased ^1^O_2_ photogeneration during sample irradiation.

Interestingly, compound **3a** showed some FIPV-inactivation activity even in the non-irradiated sample (Figure 7E, black line). It is evident that the anti-FIPV activity of the perylenylethynylphenols is inducible by daylight, and even the brief exposure of the compounds to daylight during sample preparation and pipetting of the samples onto microtiter plates is sufficient to activate the photosensitizers and manifest their light-dependent antiviral activity. These results are consistent with the observed antiviral activity of **3a** and other perylenylethynylphenols under normal lighting, as described above (Figure 5D,E and Figure 6B).

To completely eliminate the influence of daylight on the anti-FIPV activity of perylenylethynylphenols, we performed a parallel experiment in a dark room. In this experiment, all manipulations, including sample preparation and plaque assays, were performed under red light (624 ± 20 nm) (Figure 7D, bottom panel). As expected, the anti-FIPV activity of **3a** (up to 10 µM) completely disappeared (Figure 7E, red line).

Finally, we examined the photocytotoxicity of **3a** and other perylenylethynylphenols, to CRFK cells. The compounds (0 to 10 µM) were added to CRFK monolayers and irradiated with blue light (465–480 nm, 30 mW/cm^2^) for 10 min (Figure 7A, top panel). After another 48-h incubation, cell viability was measured and compared with that of non-irradiated cells (controls, Figure 7A, bottom panel). Interestingly, we observed no increase in the cytotoxicity of the studied compounds in irradiated compound-treated cells (CC_50_ > 10 uM) compared with controls. The irradiation led to the increased metabolic activity of compound-treated cells (cell viabilites > 100%), which was particularly true for compounds **3b** and **3e**. It is likely that metabolically active cells, unlike viruses, are more resistant to the deleterious effects of the compounds by undergoing metabolic restructuring and replacing oxidized membrane lipids, thereby restoring the physiological rheology of cell membranes [16]. In our previous study [20], some perylenylethynyl derivatives were highly cytotoxic for Vero cells after irradiation with blue light. It is apparent that the photocytotoxicity of perylene compounds depends not only on the compound structure and its ability to generate ^1^O_2_, but also on the cell type.

Due to the unusual mechanism of antiviral action based on membrane targeting and ^1^O_2_ photogeneration, perylenylethynylphenols, together with other perylene-based compounds, represent broad-spectrum antiviral agents active against a variety of enveloped viruses. In agreement with this claim, perylenylethynyl compounds have been previously described as potent inhibitors/inactivators of numerous phylogenetically unrelated viral pathogens, such as herpes simplex virus 1 and 2 [17], influenza A virus [16], human parainfluenza virus type 3 [61], African swine fever virus [62], human respiratory syncytial virus [61], and some members of the *Flaviviridae* family (TBEV, hepatitis C virus) [16,63].

## 3. Materials and Methods

### 3.1. General Methods

3- and 2-Ethynylperylenes [55] were obtained as described. 4-Iodophenol (Fluka) was recrystallized from *n*-hexane before use. Copper (I) iodide, bis(triphenylphosphine)dichloropalladium (Aldrich, St. Louis, MO, USA), trimethylamine (Acros, Shanghai, China), and other reagents (Chimmed, Moscow, Russia) were used without additional purification. All solvents were purified according to the Armarego and Chai guide [64].

^1^H and ^13^C NMR spectra were referenced to CDCl_3_ (7.26 ppm for ^1^H and 77.16 ppm for ^13^C). ^1^H NMR coupling constants are reported in hertz (Hz) and refer to apparent multiplicities. UV spectra were recorded on a Varian Cary 100 spectrophotometer. Fluorescence spectra were recorded using a Perkin Elmer LS 55 fluorescence spectrometer. Electrospray ionization high resolution mass spectra (ESI HRMS) were recorded using a Thermo Scientific Orbitrap Exactive mass spectrometer in positive ion mode and processed with mMass 5.5.0 software. Thin-layer liquid chromatography was performed using TLC Silica gel 60 F_254_ aluminium sheets (Merck, Rahway, NJ, USA).

### 3.2. Rate of Singlet Oxygen Measurement

The ROS generation rate was estimated using the spectrofluorimetric method, based on the fluorescense changes of Singlet Oxigen Sensor Green (SOSG^®^, Lumiprobe, Cockeysville, MD, USA) in methanol solution. Oxidation of SOSG by singlet oxygen results in peroxide formation enhancing SOSG fluorescence.

Spectrophotometric measurements were performed in a Qpod 2e thermostated cuvette holder (Quantum Northwest, Liberty Lake, WA, USA) at 25 °C and with magnetic stirring (500 rpm). Absorption spectra were recorded using a MayaPro spectrophotometer (Ocean Optics, Orlando, FL, USA) and a stabilized white light source with a SLS201L tungsten lamp (Thorlabs, Newton, NJ, USA). The fluorescence of SOSG was measured with a Flame CCD spectrometer (Ocean Optics, Orlando, FL, USA) in StripChart mode (530 nm), excitation of the SOSG fluorescence was carried out with a PLS-510 LED laser (InTop, St. Petersburg, Russia) in CW mode at the wavelength of 510 nm.

To study photosensitized ^1^O_2_ generation, we used a white MCWHLP1 LED (Thorlabs, USA) with filters to limit the radiation to the 430–450 nm range (5.5 mW/cm^2^). Illumination was uniform over the entire volume of the cuvette, to prevent artifacts associated with the diffusion of non-reacted components from entering into the illuminated volume of the cuvette. Illumination was performed in pulsed mode, with 1 s of illumination followed by 5 s of dark adaptation, during which the fluorescence spectrum of the photosensitizer-SOSG solution was recorded.

Singlet oxygen generation quantum yield for compounds **3a**–**3f** in methanol was calculated according to Equation (1):

(1) $$\varphi_{\Delta }=\varphi_{\Delta }^{0}*\frac{r}{r^{0}}\frac{\left(1-10^{{-A}^{0}}\right)}{\left(1-10^{-A}\right)},$$

where r is the rate of SOSG fluorescence increase in solution of the photosensitizer (PS), A is the PS absorbance in the region of illumination, and index 0 represents reference PS (we used perylene compound **C1T11** [65] with $\varphi_{\Delta }^{0}=0.59$ in methanol [20]).

**General procedure for the synthesis of (perylen-3-ylethynyl)-(3a–c) and (perylen-2-ylethynyl)-(3d–f) arenes.** 3- or 2-Ethynylperylene (1.2 eq.), the corresponding iodophenol (1 eq., see Table 1), bis(triphenylphosphine)dichloropalladium (0.05 eq.), and copper(I) iodide (0.1 eq.) were dissolved in dry DMF (~100 mL). The mixture was evacuated and purged with argon 5 times, then triethylamine (5 eq.) was added. The reaction mixture was heated up to 80 °C and stirred overnight. Then, the mixture was diluted with ethyl acetate (150 mL), washed with 1% aq. EDTA (200 mL), water (3 × 200 mL), and brine (200 mL), dried over anhydrous sodium sulphate, filtered off, and evaporated in vacuo to give a raw solid. The residue was purified by column chromatography in the appropriate solvent system on silica gel. The corresponding fractions were pooled and evaporated to yield desired compounds as colored solids.

**4-(Perylen-3-ylethynyl)phenol (3a).** ^1^H and ^13^C NMR and HRMS had been described before [55]. UV-Vis (96% EtOH, λ_max_, nm): 228, 257, 334, 438 and 466. Fluorescence (96% EtOH, λ_max_, nm) (ex., for em. at 520 nm): 258, 334, 440, 468; (em., for ex. 420 nm): 476, 510, 539.

**3-(Perylen-3-ylethynyl)phenol (3b)** was prepared from 3-iodophenol (220 mg, 1 mmol) and 3-ethynylperylene, purified in 0→2% EtOAc in DCM, yield 241 mg (65%). Orange solid. *R*_f_ 0.40 (DCM); dec. > 200 °C (toluene). **^1^**H NMR (500 MHz, DMSO-*d*_6_) δ 9.74 (s, 1H), 8.45 (d, *J* = 7.5 Hz, 1H), 8.40 (s, 1H), 8.37–8.29 (m, 2H), 8.00 (s, 1H), 7.88–7.74 (m, 3H), 7.62–7.50 (m, 3H), 7.27 (t, *J* = 7.9 Hz, 1H), 7.08 (d, *J* = 7.3 Hz, 1H), 7.02 (s, 1H), 6.91–6.82 (m, 1H). ^13^C NMR (126 MHz, DMSO-*d*_6_) δ 157.34, 134.17, 134.08, 131.14, 130.69, 130.67, 130.45, 129.91, 129.83, 129.79, 129.45, 128.46, 128.43, 128.18, 128.14, 127.75, 127.67, 127.64, 127.56, 127.52, 127.34, 126.81, 126.76, 123.06, 122.38, 122.28, 121.38, 120.92, 120.68, 117.81, 117.79, 116.38, 89.88, 88.85. APCI HRMS *m*/*z* 369.1286 [M+H]^+^ (calcd. for C_28_H_17_O^+^, 369.1274). UV-Vis (96% EtOH, λ_max_, nm): 220, 257, 330, 435 and 463. Fluorescence (96% EtOH, λ_max_, nm) (ex., for em. at 520 nm): 256, 330, 437, 463; (em., for ex. 420 nm): 472, 505, 537.

**4-(Perylen-3-ylethynyl)-2-bromophenol (3c)** was prepared from 2-bromo-4-iodophenol (150 mg, 0.33 mmol) and 3-ethynylperylene, purified in toluene, yield 95 mg (64%). Red solid. *R*_f_ 0.70 (DCM); dec. > 200 °C (toluene). ^1^H NMR (500 MHz, DMSO-*d*_6_) δ 10.88 (s, 1H), 8.44 (d, *J* = 7.5 Hz, 1H), 8.42–8.30 (m, 3H), 8.24 (d, *J* = 8.2 Hz, 1H), 7.91–7.85 (m, 1H), 7.86–7.78 (m, 2H), 7.75 (d, *J* = 7.8 Hz, 1H), 7.68 (t, *J* = 7.9 Hz, 1H), 7.59–7.50 (m, 3H), 7.04 (d, *J* = 8.4 Hz, 1H). ^13^C NMR (126 MHz, DMSO-*d*_6_) δ 155.12, 135.76, 134.17, 133.74, 132.26, 131.08, 130.96, 130.87, 130.12, 129.84, 128.56, 128.28, 127.85, 127.76, 127.60, 126.96, 126.92, 125.75, 121.53, 121.35, 121.21, 120.26, 119.49, 116.56, 114.27, 109.45, 94.56, 86.81. APCI HRMS *m*/*z* 447.0386 [M+H]^+^ (calcd. for C_28_H_16_BrO^+^, 447.0379). UV-Vis (96% EtOH, λ_max_, nm): 229, 257, 335, 438, and 466. Fluorescence (96% EtOH, λ_max_, nm) (ex., for em. At 520 nm): 258, 335, 439, 468; (em., for ex. 420 nm): 475, 508, 539.

**4-(Perylen-2-ylethynyl)phenol (3d)** was prepared from 4-iodophenol (220 mg, 1.0 mmol) and 2-ethynylperylene, purified in 0→2% EtOAc in DCM, yield 204 mg (55%). Brownish solid. *R*_f_ 0.36 (DCM); dec. > 200 °C (toluene). ^1^H NMR (500 MHz, DMSO-*d*_6_) δ 9.97 (s, 1H), 8.42 (d, *J* = 7.5 Hz, 1H), 8.37–8.29 (m, 3H), 7.94 (s, 1H), 7.84–7.73 (m, 3H), 7.58–7.50 (m, 3H), 7.48 (d, *J* = 8.3 Hz, 2H), 6.86 (d, *J* = 8.3 Hz, 2H).^13^C NMR (126 MHz, DMSO-*d*_6_) δ 158.13, 134.16, 134.13, 133.04, 131.02, 130.41, 130.15, 129.95, 129.50, 128.37, 128.11, 127.75, 127.57, 127.46, 127.11, 126.76, 122.34, 121.26, 121.15, 120.86, 115.75, 112.37, 90.49, 87.52. APCI HRMS *m*/*z* 369.1285 [M+H]^+^ (calcd. For C_28_H_17_O^+^, 369.1274). UV-Vis (96% EtOH, λ_max_, nm): 228, 259, 337, 295, 390, 412, and 438. Fluorescence (96% EtOH, λ_max_, nm) (ex., for em. At 520 nm): 258, 296, 337, 390, 413, 439, 467; (em., for ex. 420 nm): 442, 471, 503.

**3-(Perylen-2-ylethynyl)phenol (3e)** was prepared from 3-iodophenol (220 mg, 1.0 mmol) and 2-ethynylperylene, purified in 0→2% EtOAc in DCM, yield 179 mg (49%). Brownish solid. *R*_f_ 0.42 (DCM); dec. > 200 °C (toluene). ^1^H NMR (500 MHz, DMSO-*d*_6_) δ 9.74 (s, 1H), 8.45 (d, *J* = 7.5 Hz, 1H), 8.40 (s, 1H), 8.37–8.29 (m, 2H), 8.00 (s, 1H), 7.88–7.74 (m, 3H), 7.62–7.50 (m, 3H), 7.27 (t, *J* = 7.9 Hz, 1H), 7.08 (d, *J* = 7.3 Hz, 1H), 7.02 (s, 1H), 6.91–6.82 (m, 1H). ^13^C NMR (126 MHz, DMSO-*d*_6_) δ 157.34, 134.17, 134.08, 131.14, 130.69, 130.67, 130.45, 129.91, 129.83, 129.79, 129.45, 128.46, 128.43, 128.18, 128.14, 127.75, 127.67, 127.64, 127.56, 127.52, 127.34, 126.81, 126.76, 123.06, 122.38, 122.28, 121.38, 120.92, 120.68, 117.81, 117.79, 116.38, 89.88, 88.85. APCI HRMS *m*/*z* 369.1282 [M+H]^+^ (calcd. For C_28_H_17_O^+^, 369.1274). UV-Vis (96% EtOH, λ_max_, nm): 226, 261, 283, 322, 333, 389, 412, and 438. Fluorescence (96% EtOH, λ_max_, nm) (ex., for em. at 520 nm): 258, 283, 334, 390, 412, 439; (em., for ex. 420 nm): 440, 470, 504.

**4-(Perylen-2-ylethynyl)-2-bromophenol (3f)** was prepared from 2-bromo-4-iodophenol (150 mg, 0.33 mmol) and 2-ethynylperylene, purified in toluene, yield 116 mg (77%). Yellow solid. *R*_f_ 0.72 (DCM); dec. > 200 °C (toluene). ^1^H NMR (500 MHz, DMSO-*d*_6_) δ 10.87 (s, 1H), 8.44–8.28 (m, 4H), 7.94 (s, 1H), 7.84–7.71 (m, 4H), 7.56–7.45 (m, 4H), 7.04 (d, *J* = 8.4 Hz, 1H).^13^C NMR (126 MHz, DMSO-*d*_6_) δ 155.09, 135.86, 134.24, 134.15, 132.16, 131.15, 130.50, 130.42, 129.99, 129.56, 128.53, 128.25, 127.81, 127.74, 127.60, 127.31, 126.86, 122.49, 121.39, 121.36, 121.00, 120.88, 116.58, 114.17, 109.38, 88.88, 88.65. APCI HRMS *m*/*z* 447.0390 [M+H]^+^ (calcd. for C_28_H_16_BrO^+^, 447.0379). UV-Vis (96% EtOH, λ_max_, nm): 233, 258, 295, 326, 391, 412, and 438. Fluorescence (96% EtOH, λ_max_, nm) (ex., for em. at 520 nm): 258, 295, 337, 391, 413, 439, 469; (em., for ex. 440 nm): 442, 471, 503.

### 3.3. Biological Studies

#### 3.3.1. Cells and Viruses

SARS-CoV-2 (strain SARS-CoV-2/human/Czech Republic/951/2020) was isolated from a clinical specimen at the National Institute of Health, Prague, Czech Republic, and was kindly provided by Dr. Jan Weber, Institute of Organic Chemistry and Biochemistry, Prague, Czech Republic. In our antiviral and mechanistic assays, we also used FIPV (ATCC VR990). Experiments using authentic SARS-CoV-2 and FIPV were performed in our BSL-3 and BSL-2 facilities, respectively.

Vero cells (ATCC CCL-81) were used for SARS-CoV-2 propagation and for anti-SARS-CoV-2 assays, whereas Vero E6 cells (ATCC CRL-1586) were used for plaque assays. CRFK cells (ATCC CCL-94) were used for anti-FIPV assays and FIPV-based plaque assays. PS cells [66] were used for studies of penetration of the compounds into cell membranes. PS cells were provided by the National Reference Laboratory for Tissue Cultures, National Institute of Public Health, Prague, Czech Republic. Vero, Vero E6, and CRFK cells were cultured in Dulbecco’s modified Eagle’s medium (DMEM), whereas PS cells were cultured in Leibovitz’s (L-15) medium. The media were supplemented with 3% (L-15) or 10% (DMEM) newborn calf serum, 100 U/mL penicillin, 100 µg/mL streptomycin, and 1% glutamine (Sigma-Aldrich, Prague, Czech Republic). Vero and Vero E6 cells were cultured at 37 °C under 5% CO_2_, whereas PS cells were cultivated at 37 °C under a normal atmosphere (without CO_2_ supplementation).

#### 3.3.2. Cytotoxicity Assay

Vero cells were cultured for 24 h in 96-well plates to form a confluent monolayer, and then were treated with the tested compounds at concentrations of 0–10 µM. After 48 h of cultivation in the dark at 37 °C under 5% CO_2_, the cell culture medium was aspirated. The potential cytotoxicity of the tested derivatives was determined based on cell viability using Cell Counting Kit-8 (Dojindo Molecular Technologies, Munich, Germany) according to the manufacturer’s instructions (Figure 5A).

#### 3.3.3. Virus Titer Reduction Assay

Virus titer reduction assay was performed as described previously [20]. Briefly, Vero cells were seeded in 96-well plates and incubated for 24 h to form a confluent monolayer. The virus in DMEM (MOI of 0.1) was mixed with each compound (0–2 µM) and incubated in the dark at 37 °C for 30 min and then used for infection of the cells. At 48 h post-infection (p.i.), the culture medium was collected and viral titers were determined by plaque assays (expressed as PFU/mL) (Figure 5B) as previously described [67] and used to estimate the 50% effective concentration (EC_50_) (Table 1). We also calculated EC_50_ values from log-transformed virus titers (App. EC_50_) (Supplementary Table S1) to better visualize the biological activity of the compounds.

#### 3.3.4. Determination of the Virucidal (Virus-Inactivating) Activity of the Compounds

To determine the virucidal activity of compound **3a** (10 µM), SARS-CoV-2 in DMEM (titers of 10^7^, 10^6^, and 10^4^ PFU/mL) was mixed with the compound and incubated in the dark at 37 °C for 120 min. Subsequently, the viability of the compound-treated virus was estimated using a plaque assay, as previously described [67]. Viral titers were expressed as PFU/mL (Figure 6A).

#### 3.3.5. Confocal Microscopy

Confocal microscopy was used to study the penetration of compound **3a** into cellular membranes. PS cells were treated with **3a** (10 µM) in a µ-Slide 8 Well (IbidiGmbH, Gräfelfing, Germany) in the dark for 60 min at 37 °C. The samples were analyzed for fluorescent signal distribution and intensity using a Leica SP8 confocal microscope (Leica, Wetzlar, Germany) as described previously [20].

#### 3.3.6. Interaction of the Compounds with Liposomes

The samples’ steady-state fluorescence characteristics were measured in L-format using a Chronos DFD Fluorescence spectrometer (ISS, Baltimore, MD, USA) equipped with a 300 W Cermax xenon arc lamp (ISS, Baltimore, MD, USA), a concave holographic grating monochromator, and a PMT detector. The required amount of each sample was diluted in DMSO and measured in a 1-cm quartz cuvette, at a constant temperature of 25 °C. The resulting data were evaluated using Vinci software version 2 (ISS, Baltimore, MD, USA) and correlated to the utilized optical configuration.

The kinetics of the incorporation of compounds into liposome membrane models were determined using steady-state fluorescence spectroscopy at a constant excitation and emission wavelength, according to the corresponding sample excitation and emission maxima. We added 50 μL of LNP suspension to 10 µM of the analyte in PBS and monitored the increase in fluorescence intensity over the time range 0–2500 s.

#### 3.3.7. Studies of Photodynamic Inactivation of FIPV Virions

Virus in DMEM (titer of 10^6^ PFU/mL) was mixed with **3a** (0–10 µM) in a microtiter plate in daylight and irradiated for 10 min at RT with LEDs (465–480 nm) at an approximate power density of 30 mW/cm^2^ (Figure 7D, top panel). As a negative control, the virus was mixed with **3a** (0–10 µM) in daylight and incubated with the compound for 10 min in the dark at RT (Figure 7D, middle panel). Subsequently, both irradiated and non-irradiated virus samples were incubated in the dark at 37 °C for an additional 60 min. Viral titers were determined by plaque assays (also in daylight). To eliminate the influence of daylight on compound activity, the entire experiment, including all manipulations with the samples, was performed in a dark room under red light only. The virus sample was mixed with **3a** (0–10 µM), incubated at 37 °C for 60 min, and the viability of the virus was assessed by plaque assays (Figure 7D, bottom panel). The plaque assays were also performed under red light.

#### 3.3.8. Studies of Light-Induced Cytotoxicity (Photocytotoxicity)

To determine the light-induced cytotoxicity of the compounds, CRFK cells were cultured in 96-well plates for 24 h to form a confluent monolayer and then treated with the tested compounds at concentrations ranging from 0 to 10 µM in daylight. Subsequently, cells treated with the compounds were irradiated with LEDs (465–480 nm, 30 mW/cm^2^) for 10 min at RT (Figure 7A, top panel). As a negative control, CRFK cells were treated with the compounds in daylight and then incubated in the dark at RT for 10 min (Figure 7A, bottom panel). Subsequently, both irradiated and non-irradiated cell monolayers were incubated for 48 h in the dark at 37 °C. After incubation, the potential photocytotoxicity of the compounds was determined using Cell Counting Kit-8 (Dojindo Molecular Technologies, Munich, Germany) according to the manufacturer’s instructions.

## 4. Conclusions

In conclusion, this study investigated the spectral properties and ^1^O_2_ generation capability of a series of novel perylene-based compounds. Antiviral activity against two important coronaviruses, SARS-CoV-2 and FIPV, was studied in vitro to elucidate their mechanism of antiviral action based on (i) specific interaction with the viral envelope and (ii) photosensitization and ^1^O_2_-mediated impairment of viral particles. The results revealed that 3-ethynylperylene derivatives exhibited higher antiviral activity compared to 2-ethynylperylene derivatives. Compound **3b** showed the strongest potency against SARS-CoV-2 in the whole series. Interestingly, the presence of a bromine atom in the compounds did not significantly affect their antiviral activity.

Analysis of singlet oxygen generation and antiviral activity data suggests that the differences in antiviral properties among perylene photosensitizers could not be attributed solely to variations in their singlet oxygen generation capacity. However, a trend was observed, indicating that compounds with a higher quantum yield of singlet oxygen generation generally exhibited higher antiviral activity. These findings highlight the importance of both structural factors and affinity to the lipid bilayer for determining the antiviral activity of perylene derivatives.

The anti-SARS-CoV-2 and anti-FIPV activities of the perylenylethynylphenols were strictly dependent on the excitation light and disappeared when the experiments were performed under red light (with a wavelength far from the excitation maxima of the compounds). Exposure of the virus–compound mixture to daylight (normal lighting conditions) during sample preparation was sufficient for the induction of the light-dependent antiviral activity of the perylenylethynylphenols. Thus, all of the observed antiviral effects of these compounds were induced exclusively by daylight and were even enhanced by light of the excitation wavelength. The light-dependent antiviral activity of perylenylethynylphenols is closely related to ^1^O^2^ photogeneration. In fact, ^1^O_2_ itself is responsible for the antiviral (or virucidal/virus-inactivating) activity of the compounds via the peroxidation of membrane lipids and destruction of viral envelopes, leading to blockage of the virus-cell fusion machinery.

Perylenylethynylphenols and other amphipathic perylene compounds hold promise as potential candidates for the development of effective antiviral agents against enveloped viruses. Further research is warranted to explore the scope and limitations of these compounds, paving the way for their potential application in the field of antiviral therapeutics.

## Figures and Tables

**Table 1:** Spectral properties, singlet oxygen generation, and biological evaluation of perylenylethynylphenols **3a**–**f**.

Compound	Structure	^abs^λ_max_, nm	^em^λ_max_, nm	ϕ_Δ_(^1^O_2_)	SARS-CoV-2 EC_50_ (μM) ^a,b^	95% CI	Vero Cells CC_50_ (μM) ^a^	SI
**3a** (**HOPY11**)	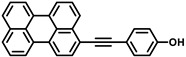	466	476	0.422	0.1203	0.085–0.171	>10	>83
**3b** (**HO3PY11**)	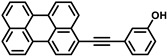	463	472	0.451	0.05109	0.039–0.067	>10	>196
**3c** (**HOBrPY11**)	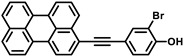	466	475	0.282	0.2274	0.150–0.346	>10	>44
**3d** (**HOPY13**)	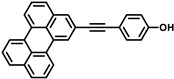	438	442	0.297	0.6138	0.394–0.956	>10	>16
**3e** (**HO3PY13**)	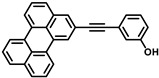	438	440	0.460	0.3472	0.234–0.514	>10	>29
**3f** (**HOBrPY13**)	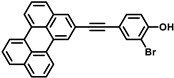	438	442	0.332	0.5290	0.165–1.700	>10	>19

^a^ Determined from three independent experiments. ^b^ Expressed as a 50% reduction in viral titer and calculated from the inflexion points of sigmoidal dose-response curves using GraphPad Prism 7.04 (GraphPad Software, Inc., La Jolla, CA, USA).

## Data Availability

Not applicable.

## Supplementary Materials

The following supporting information can be downloaded at: https://www.mdpi.com/article/10.3390/molecules28176278/s1, Figures S1–S5: NMR spectra of compounds **3b–f**, Table S1: EC_50_ values calculated from the log-transformed viral titers.

## Sample Availability

Samples of compounds **3a**–**f** are available from the authors.
