# Supplementary material for: Membrane-Targeting Perylenylethynylphenols Inactivate Medically Important Coronaviruses via the Singlet Oxygen Photogeneration Mechanism
Source: Molecules. 2023 Aug 28;28(17):6278. doi: 10.3390/molecules28176278 (PMC10488391; doi:10.3390/molecules28176278)
Supplement: Supplementary file 1 [file molecules-28-06278-s001.zip › molecules-2509330-supplementary.pdf]

Supplementary Information

Membrane-Targeting Perylenylethynylphenols Inactivate Medically Important Coronaviruses via the Singlet Oxygen Photogeneration Mechanism

Kseniya A. Mariewskaya [1], Daniil A. Gvozdev [2], Alexey A. Chistov [1], Petra Straková [3,4,5], Ivana Huvarová [3], Pavel Svoboda [3,4,5,6], Jan Kotouček [7], Nikita M. Ivanov [1], Maxim S. Krasilnikov [1,8], Mikhail Y. Zhitlov [1,8], Alexandra M. Pak [1,†], Igor E. Mikhnovets [1], Timofei D. Nikitin [1], Vladimir A. Korshun [1,*], Vera A. Alferova [1], Josef Mašek [7], Daniel Růžek [3,4,5], Luděk Eyer [3,4,5,*] and Alexey V. Ustinov [1]

1 Shemyakin-Ovchinnikov Institute of Bioorganic Chemistry, Miklukho-Maklaya 16/10, 117997 Moscow, Russia
2 Department of Biology, Lomonosov Moscow State University, Leninskie Gory 1-12, 119234 Moscow, Russia
3 Laboratory of Emerging Viral Diseases, Veterinary Research Institute, Hudcova 296/70, CZ-621 00 Brno, Czech Republic
4 Institute of Parasitology, Biology Centre of the Czech Academy of Sciences, Branišovská 1160/31, CZ-370 05 České Budějovice, Czech Republic
5 Department of Experimental Biology, Faculty of Science, Masaryk University, Kamenice 753/5, CZ-625 00 Brno, Czech Republic
6 Department of Pharmacology and Pharmacy, Faculty of Veterinary Medicine, University of Veterinary Sciences Brno, Palackého tř. 1946/1, CZ-612 42 Brno, Czech Republic
7 Department of Pharmacology and Toxicology, Veterinary Research Institute, Hudcova 296/70, CZ-621 00 Brno, Czech Republic
8 Department of Chemistry, Lomonosov Moscow State University, Leninskie Gory 1-3, 119991 Moscow, Russia
† Present address: Nesmeyanov Institute of Organoelement Compounds, 119991 Moscow, Russia
* Correspondence: v-korshun@yandex.ru (V.A.K.); ludek.eyer@vri.cz (L.E.)

Figures S1–S5: ^{1}H and ^{13}C NMR spectra of compounds **3b–3f**.
Supplementary Table S1. EC_{50} values calculated from the log-transformed viral titers.

Figure S1a. ^{1}H NMR spectrum of compound **3b**.

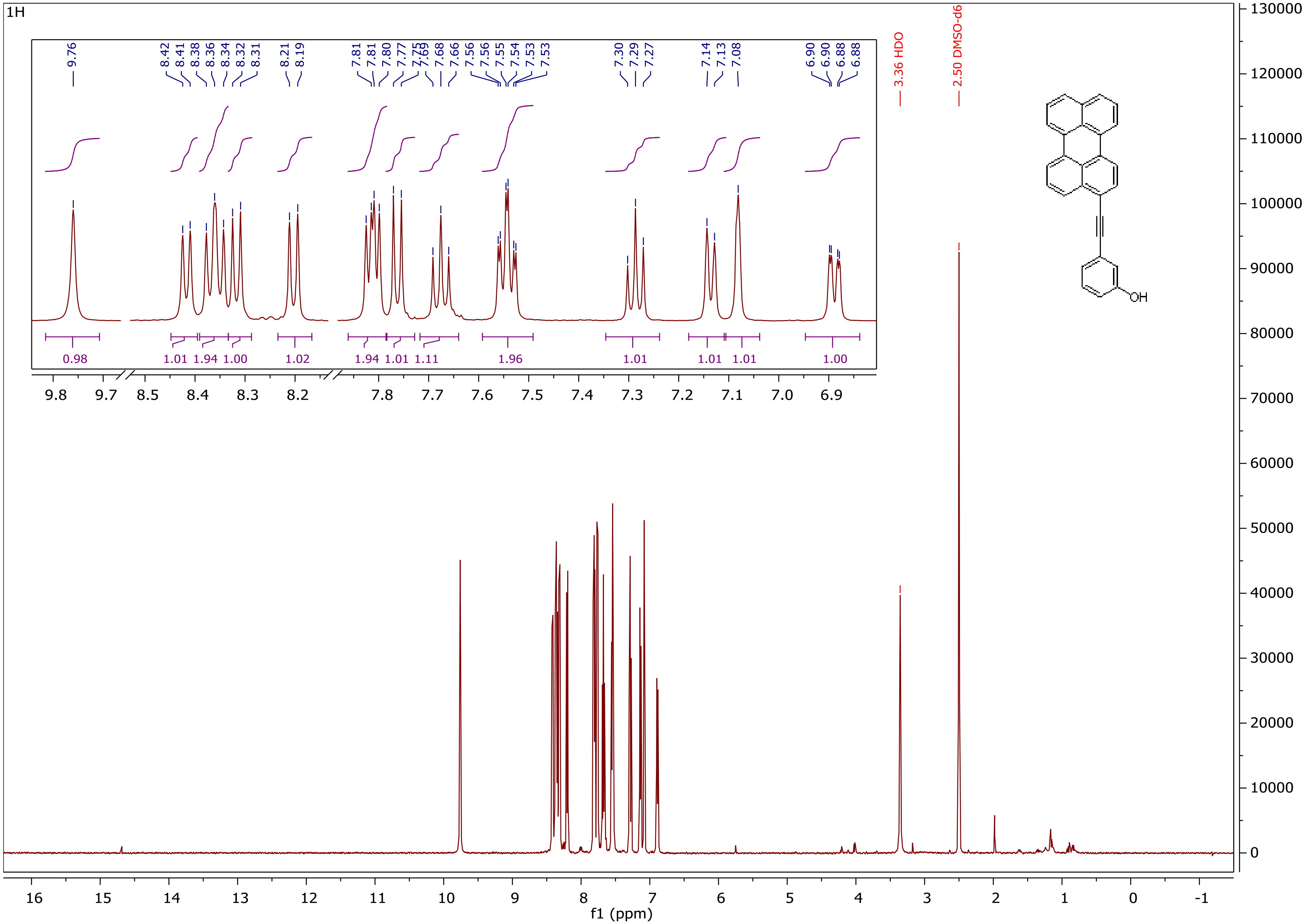

Figure S1b. ^{13}C NMR spectrum of compound **3b**.

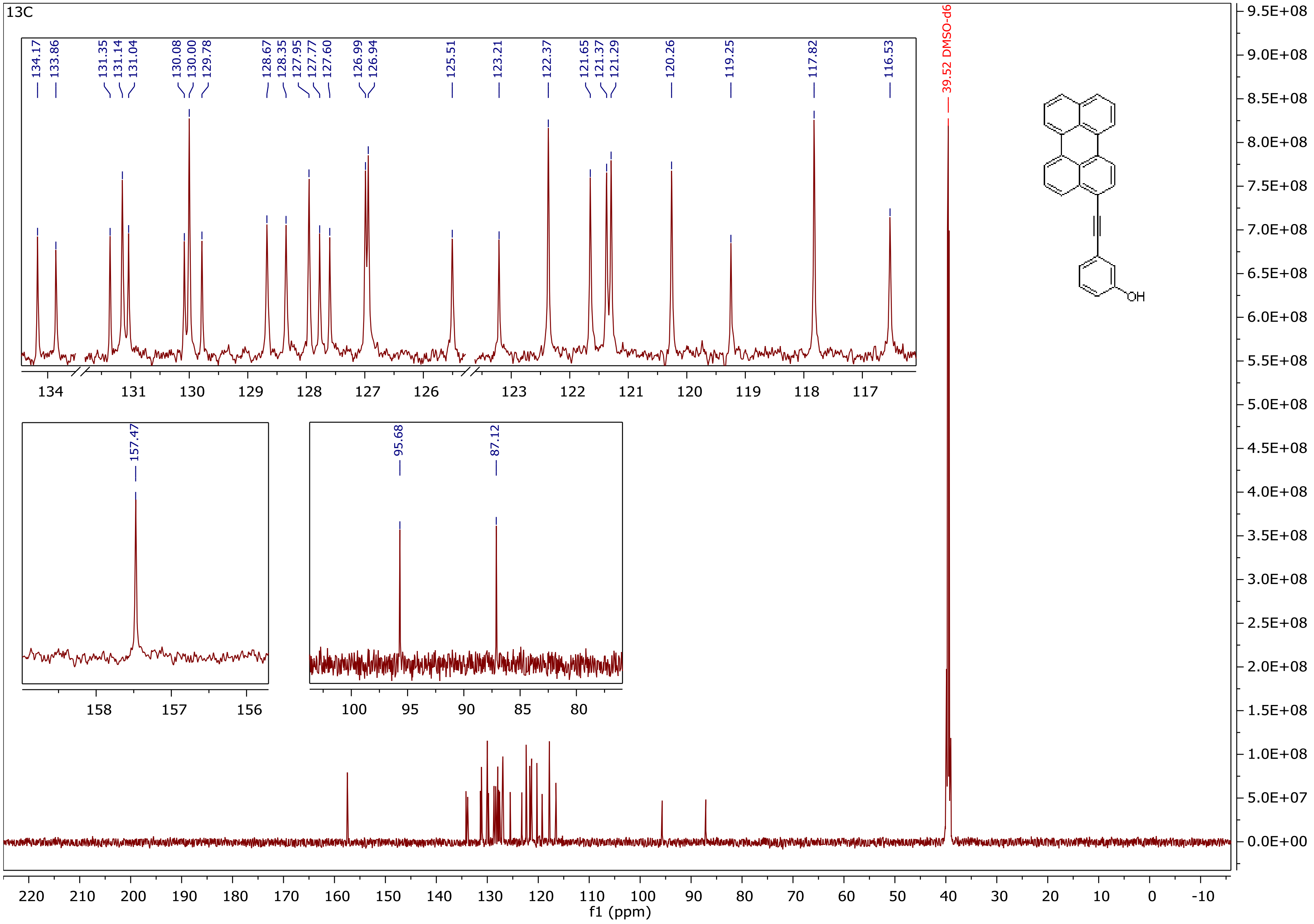

Figure S2a. ^{1}H NMR spectrum of compound **3c**.

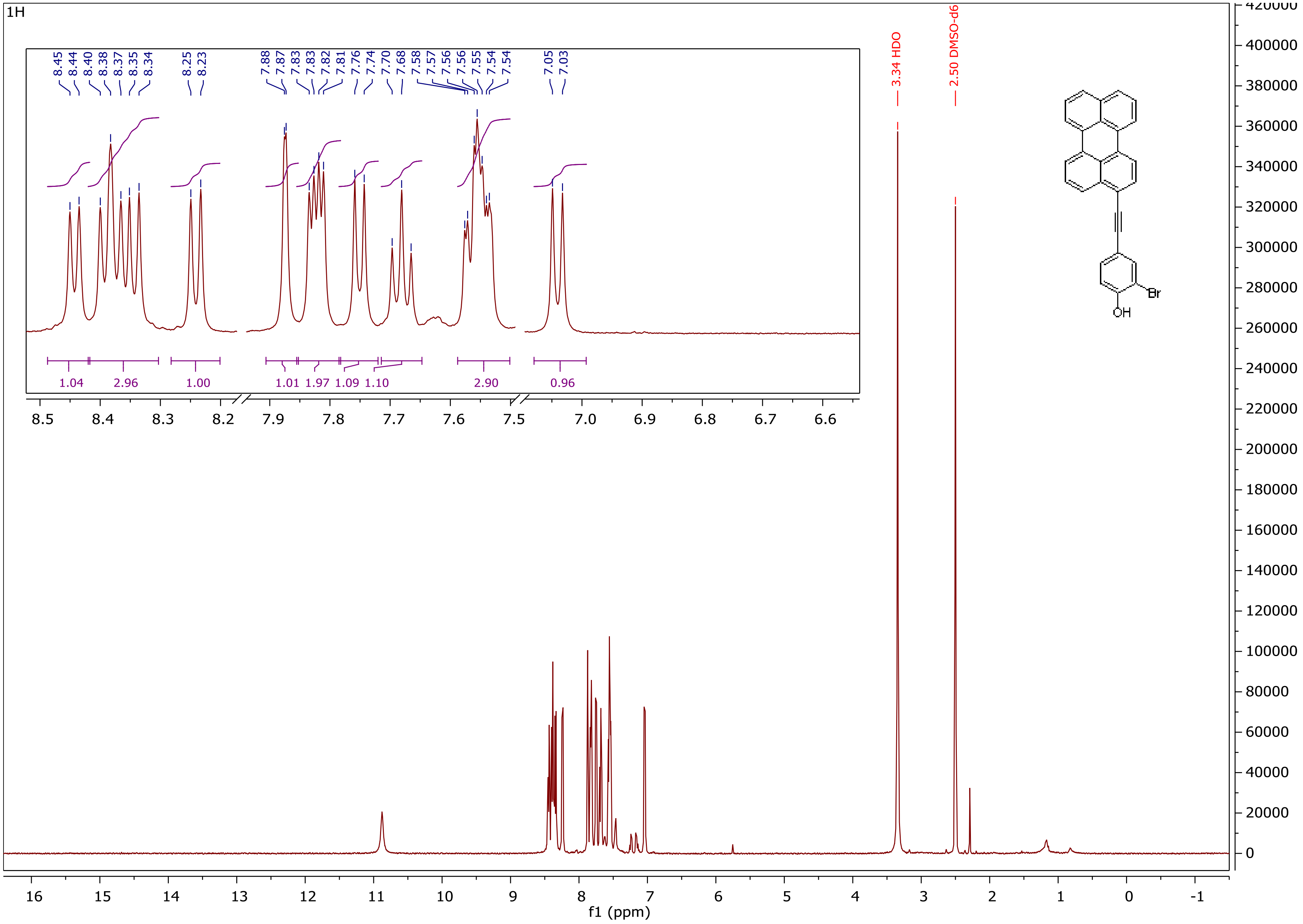

Figure S2b. ^{13}C NMR spectrum of compound **3c**.

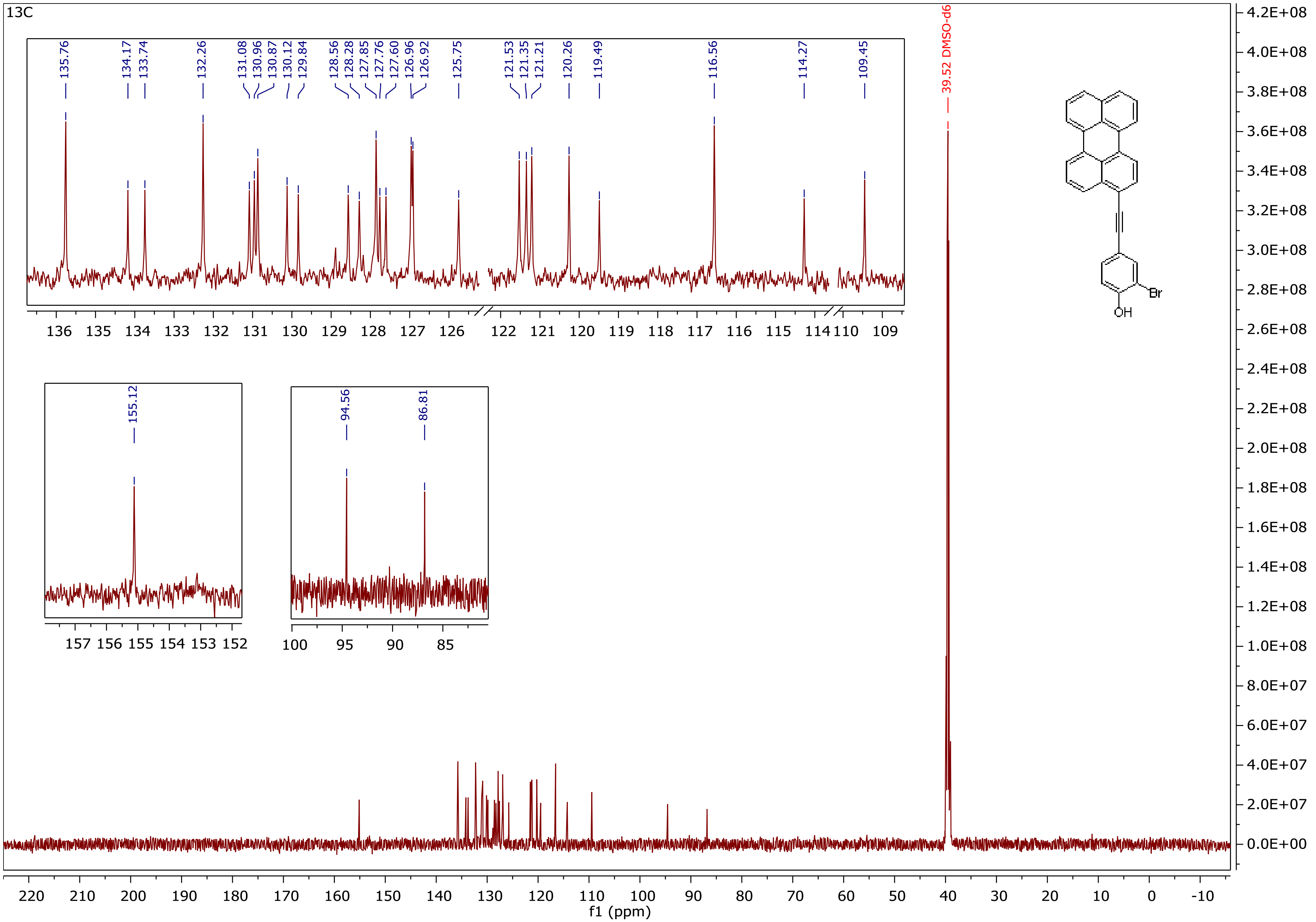

Figure S3a. ^{1}H NMR spectrum of compound **3d**.

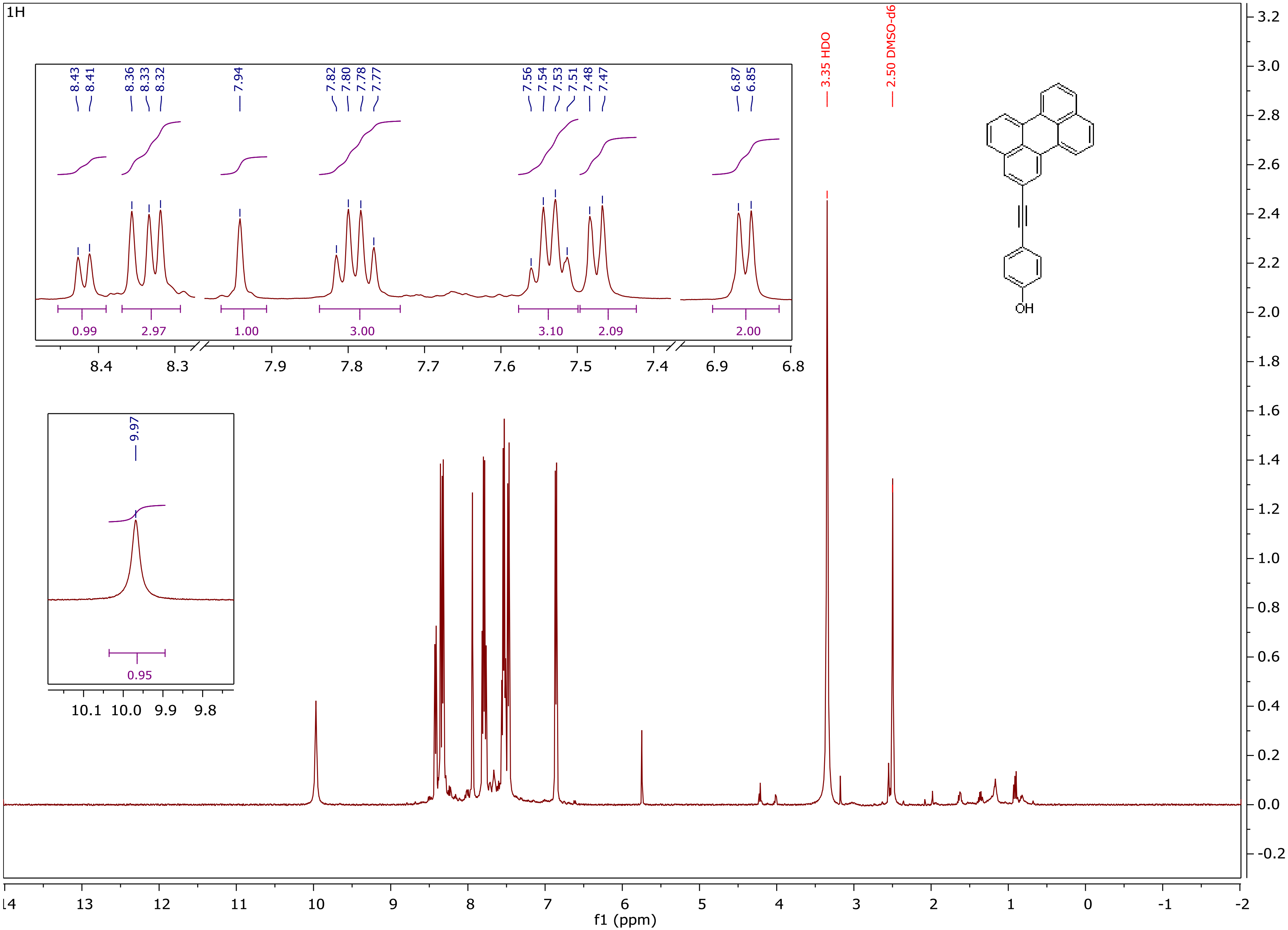

Figure S3b. ^{13}C NMR spectrum of compound **3d**.

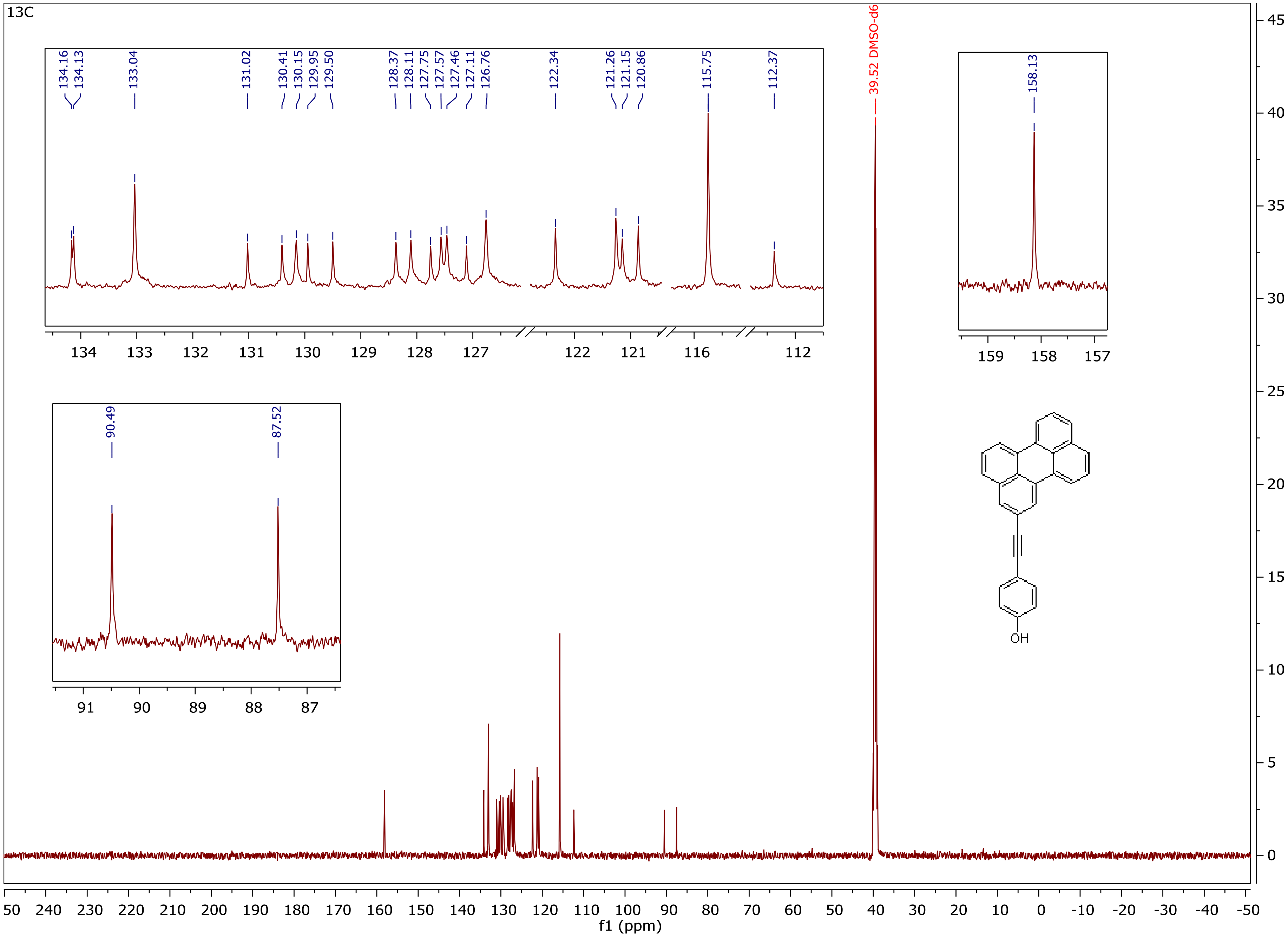

Figure S4a. ^{1}H NMR spectrum of compound **3e**.

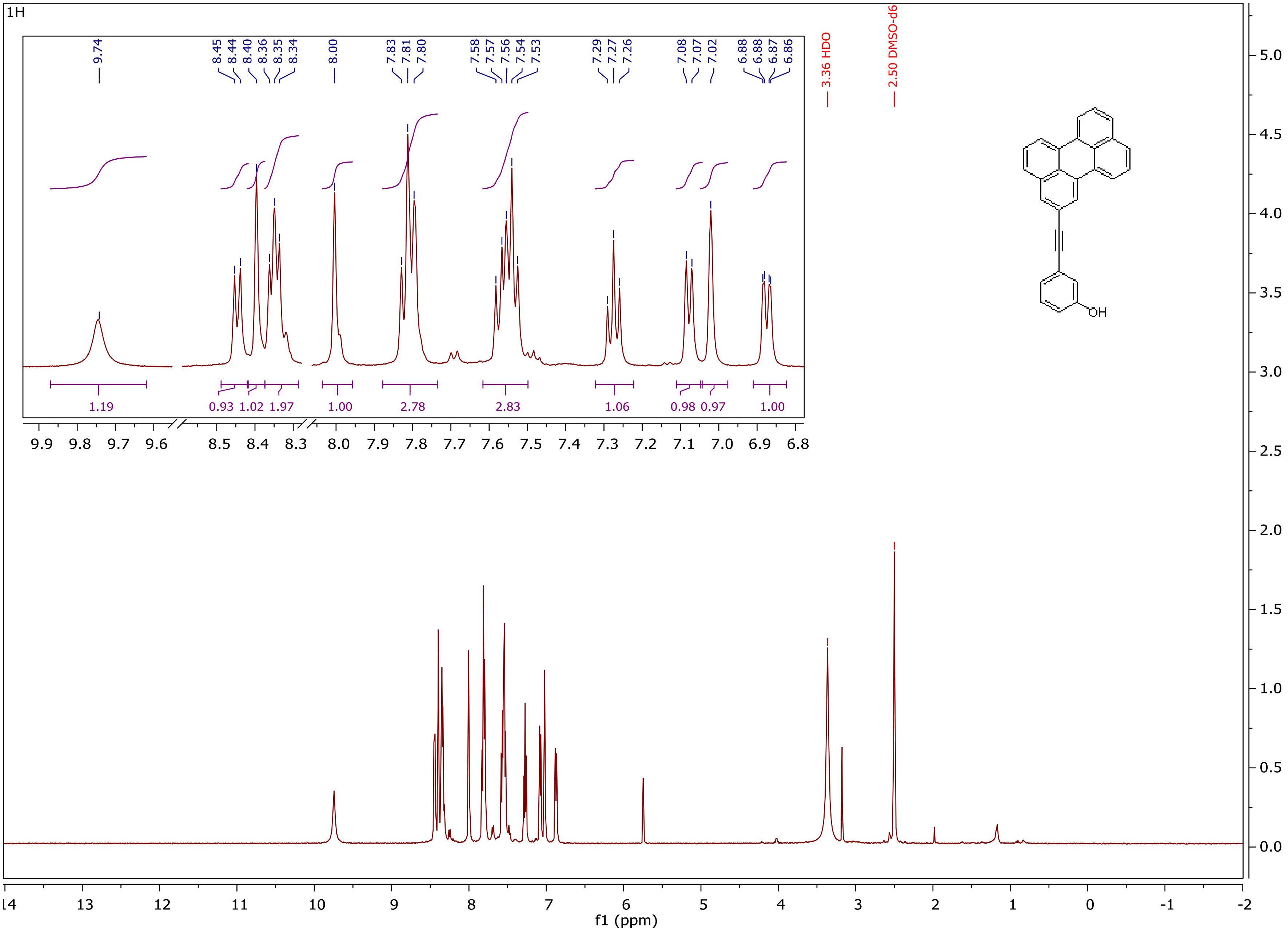

Figure S4b. ^{13}C NMR spectrum of compound **3e**.

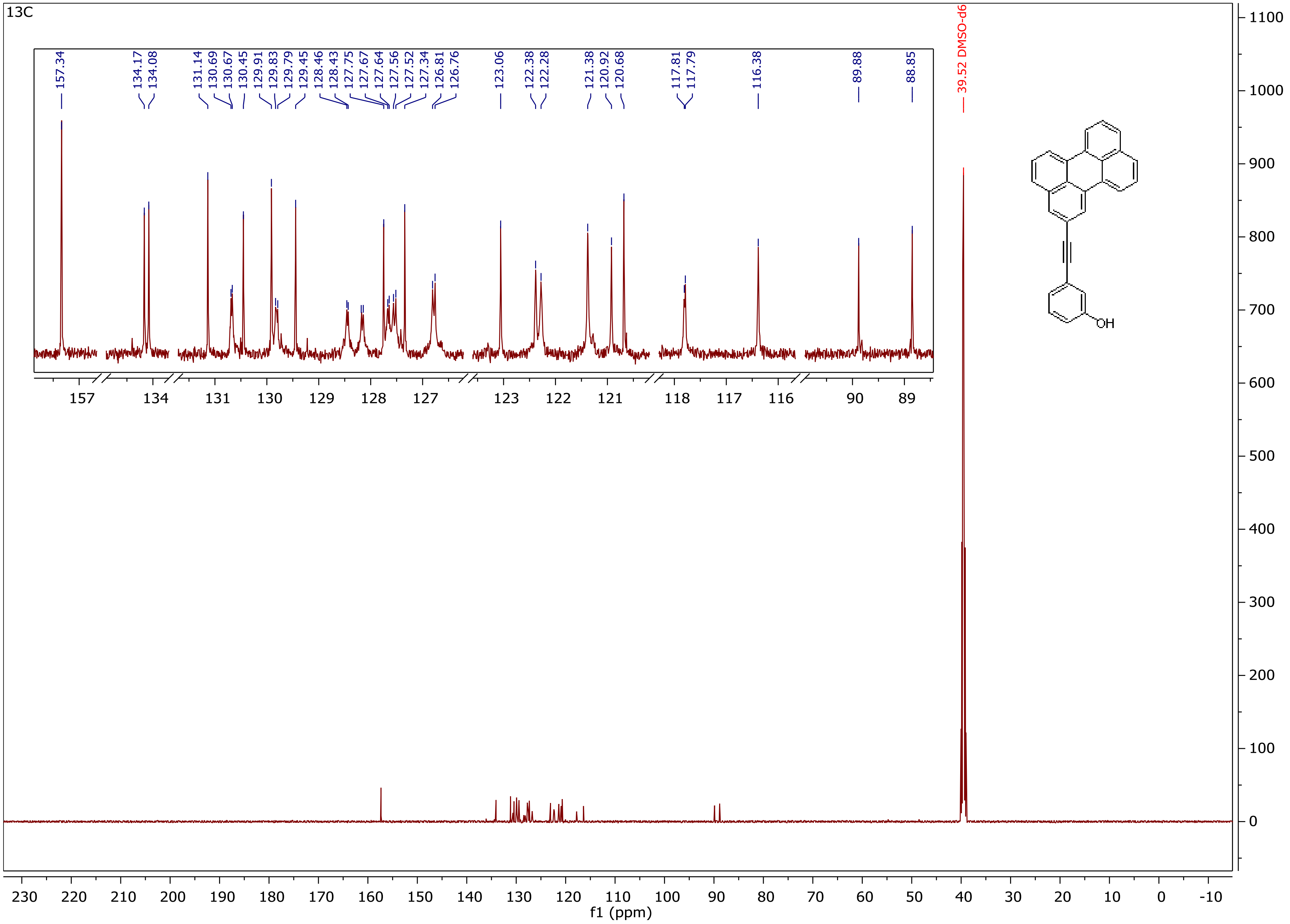

Figure S5a. ^{1}H NMR spectrum of compound **3f**.

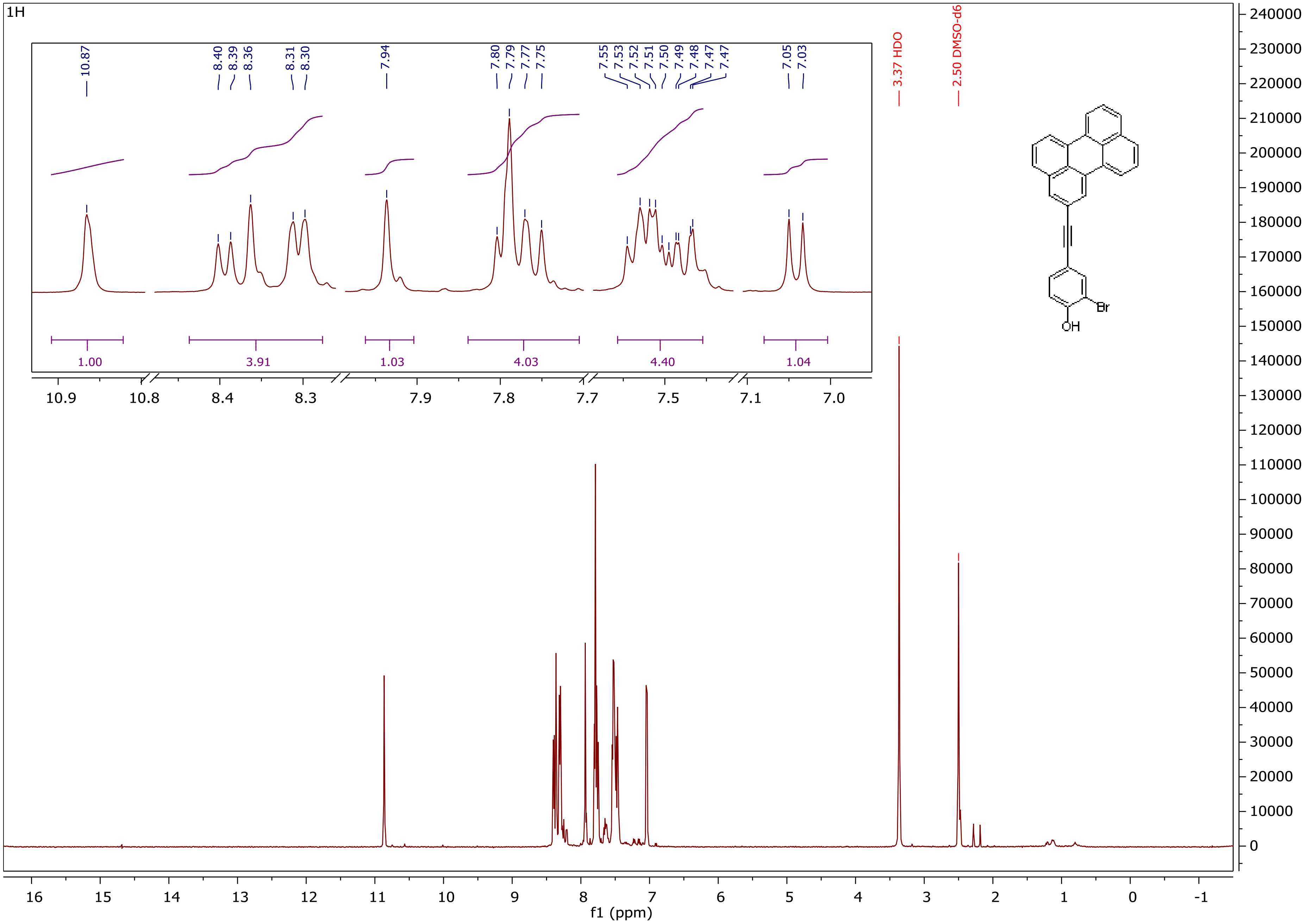

Figure S5b. ^{13}C NMR spectrum of compound **3f**.

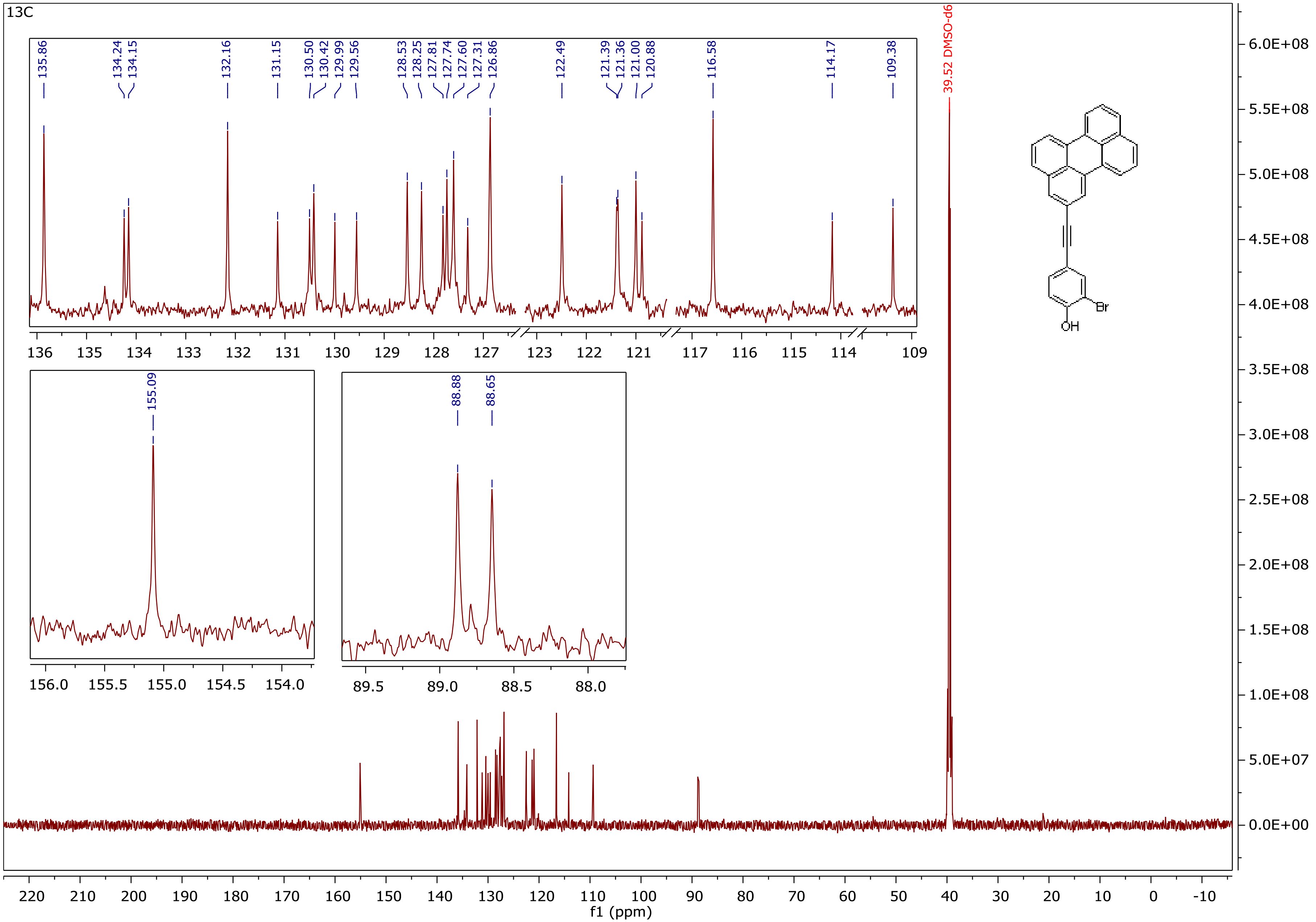

Supplementary Table S1. EC_{50} values calculated from the log-transformed viral titers.

Compound	App. EC_{50} (µM)[a,b]	95% CI
**3a**	1.786	1.563–2.041
**3b**	0.5062	0.4059–0.6312
**3c**	1.096	0.8251–1.456
**3d**	2.223	1.694–2.916
**3e**	1.575	1.314–1.889
**3f**	2.675	1.747–4.098

[a)] Determined from three independent experiments.

[b)] Expressed as a 50% reduction in viral titer and calculated from the inflexion points of sigmoidal dose-response curves, which were obtained by a nonlinear fit of log-transformed inhibitor concentrations versus normalized log-transformed response using GraphPad Prism 7.04 (GraphPad Software, Inc., USA).
